# Novelties in the pharmacological approaches for chronic heart failure: new drugs and cardiovascular targets

**DOI:** 10.3389/fcvm.2023.1157472

**Published:** 2023-06-02

**Authors:** Michele Correale, Lucia Tricarico, Francesca Croella, Simona Alfieri, Francesco Fioretti, Natale Daniele Brunetti, Riccardo M. Inciardi, Savina Nodari

**Affiliations:** ^1^ Department of Cardiothoracic, Policlinico Riuniti University Hospital, Foggia, Italy; ^2^ Department of Medical & Surgical Sciences, University of Foggia, Foggia, Italy; ^3^ Cardiology Section, Department of Medical and Surgical Specialties, Radiological Sciences and Public Health, ASST Spedali Civili Hospital and University of Brescia, Brescia, Italy

**Keywords:** new drugs, therapy targets, heart failure, cardiac metabolism, mitochondrial function, CGMP pathway

## Abstract

Despite recent advances in chronic heart failure (HF) management, the prognosis of HF patients is poor. This highlights the need for researching new drugs targeting, beyond neurohumoral and hemodynamic modulation approach, such as cardiomyocyte metabolism, myocardial interstitium, intracellular regulation and NO-sGC pathway. In this review we report main novelties on new possible pharmacological targets for HF therapy, mainly on new drugs acting on cardiac metabolism, GCs-cGMP pathway, mitochondrial function and intracellular calcium dysregulation.

## 1. Introduction

**Figure 1:**
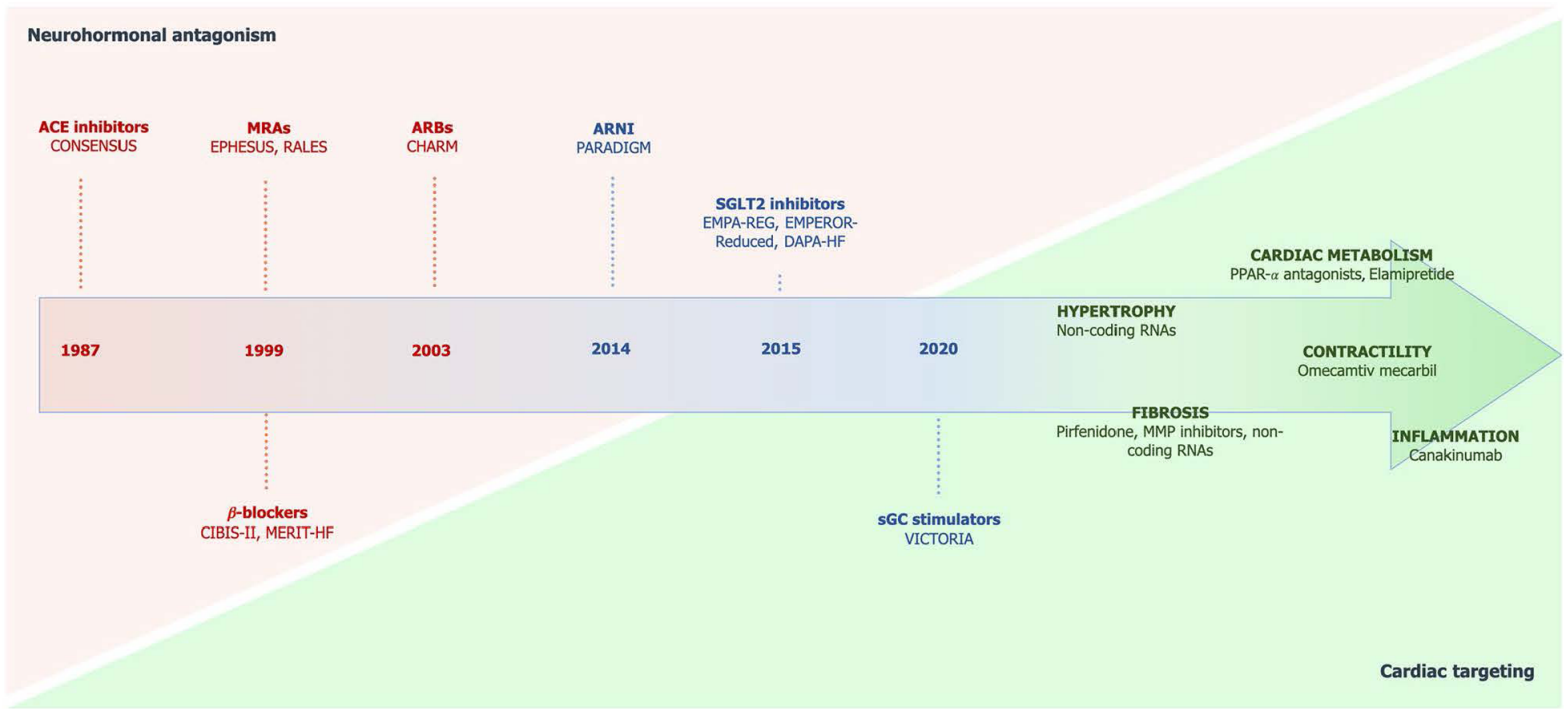
Chronologic development of drugs in HF with the shift from neurohormonal antagonism to specific cardiac targeting. Taken from Ghionzoli et al. (5). ACE, angiotensin-converting enzyme; ARBs, angiotensin receptor blockers; ARNI, angiotensin receptor neprilysin inhibitor; MRAs, mineralocorticoid receptor antagonists; sGC, soluble guanylate cyclase; SGLT2, sodium-glucose cotransporter 2.

**Figure 2:**
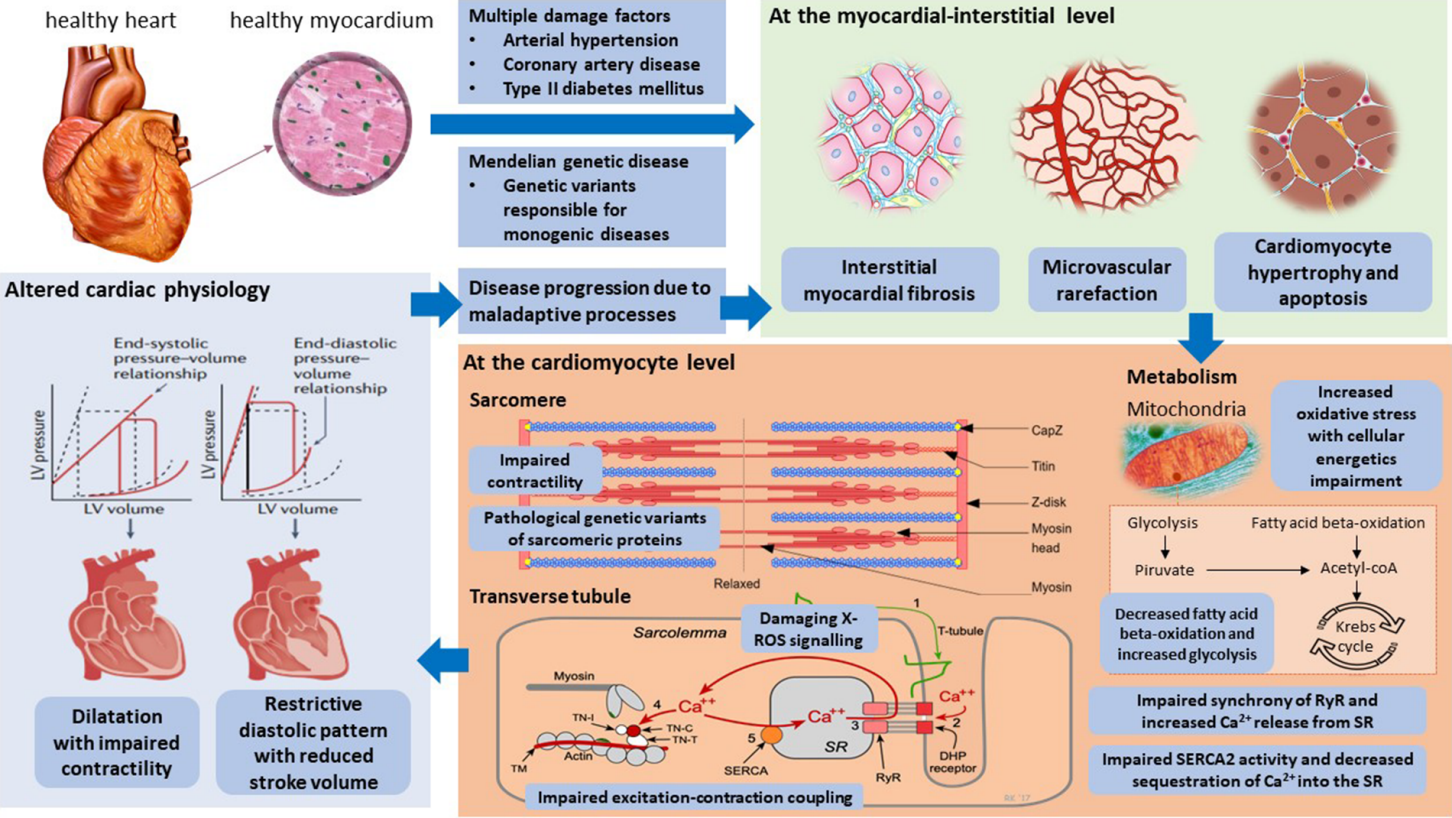
Pathophysiological mechanisms and innovative potential cardiac targets in the failing heart. Taken from Weldy et al. (6). LV, left ventricular; RYR2, ryanodine receptor 2; SERCA2, sarcoplasmic–endoplasmic reticulum Ca^2+^ ATPase 2; SR, sarcoplasmic reticulum; TCA, tricarboxylic acid.

**Figure 3:**
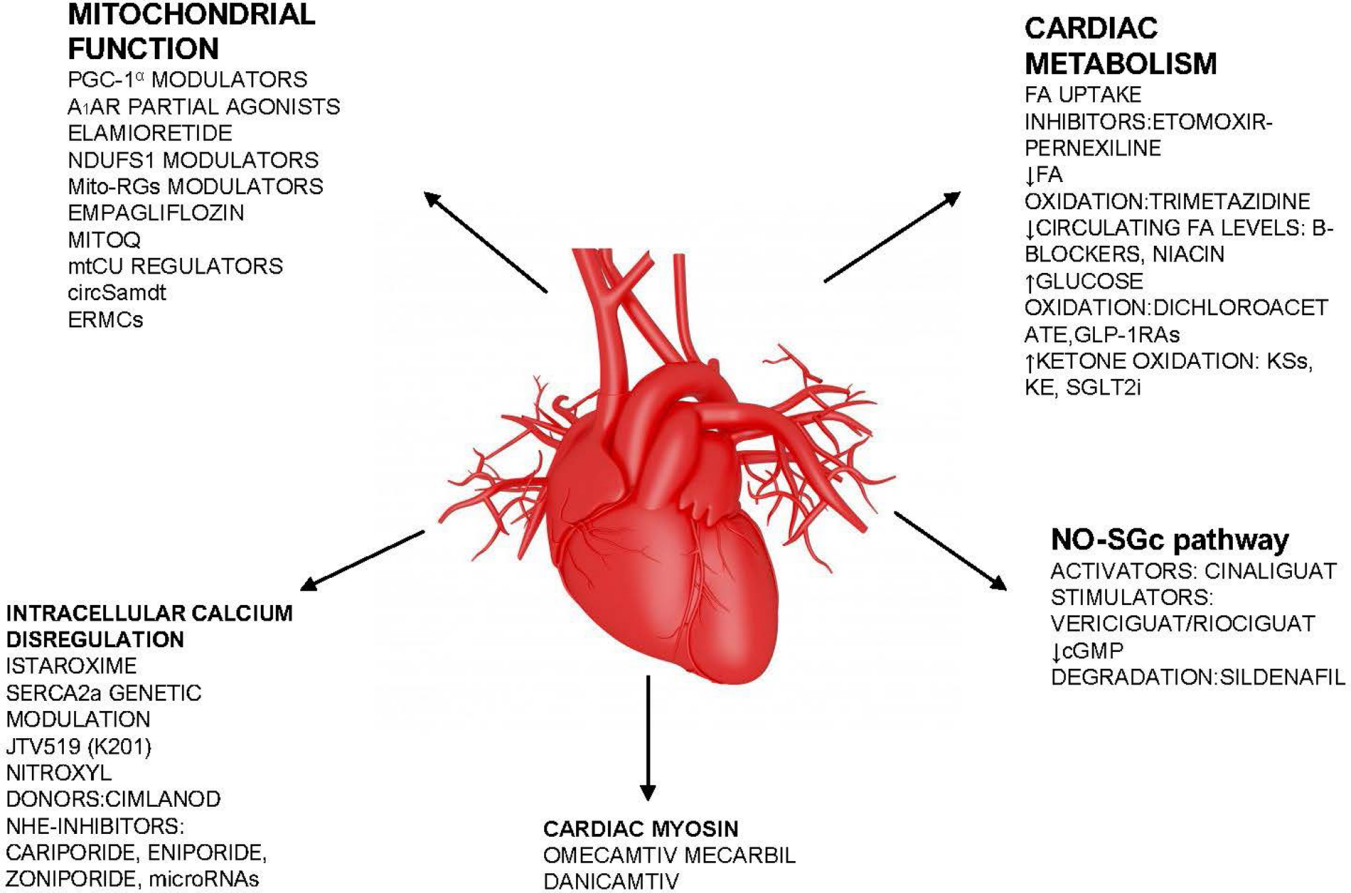
Summary figure (list of potential new drugs). FA, fatty acid; sGC, soluble guanylate cyclase; SGLT2i, sodium-glucose cotransporter 2 inhibitor; NHE-1, Na H^+^ exchanger; PGC-1α, peroxisome proliferator-activated receptor γ coactivator 1 α; Ndufs1 NADH, ubiquinone oxidoreductase core subunit S1; mtCU, Ca^2+^ uniporter; SERCA2, sarcoplasmic–endoplasmic reticulum Ca^2+^ ATPase 2; mPTP, mitochondrial permeability transition pore; A1AR, adenosine A1 receptor; MitoQ, mitoquinone.

**Figure 4:**
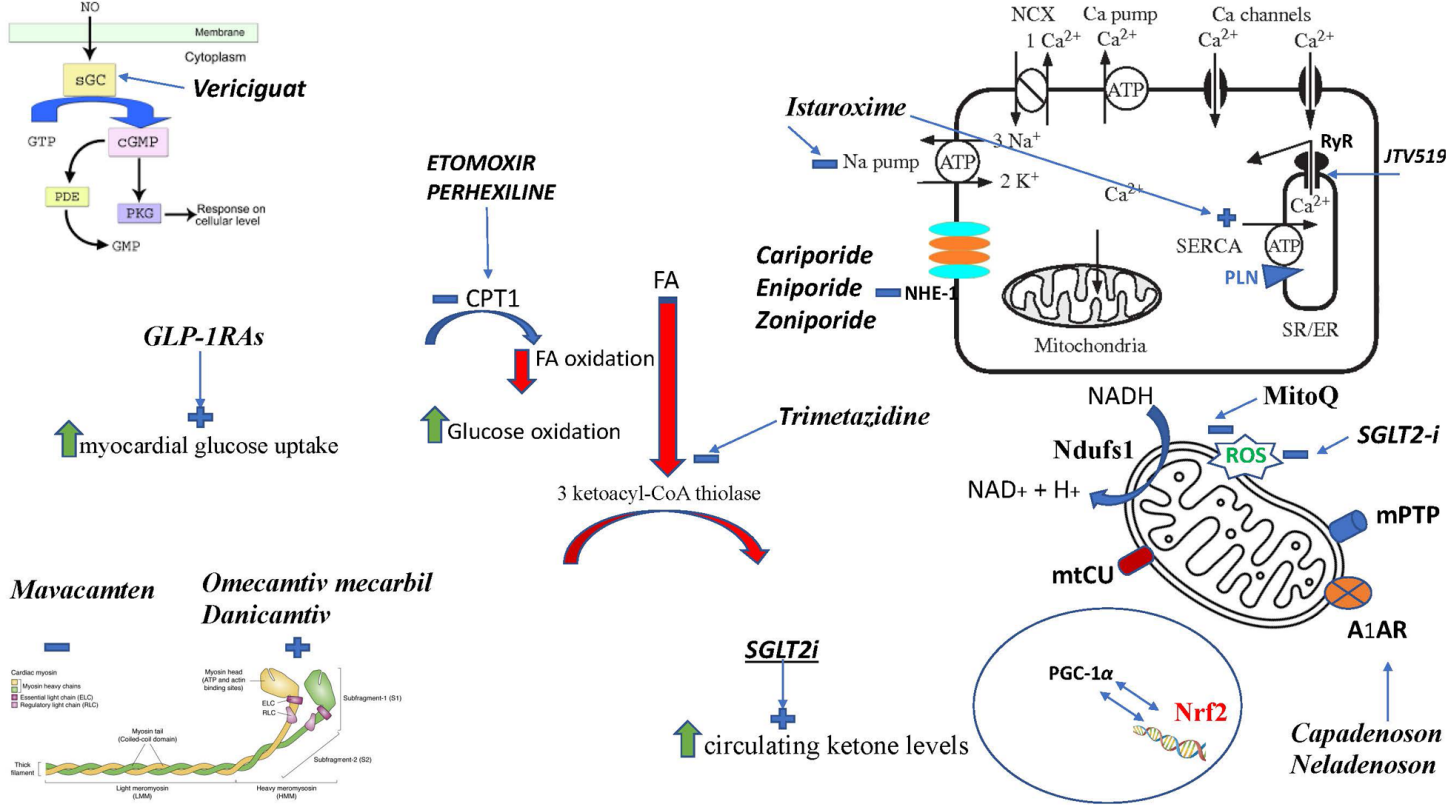
Figure summarizing the mechanism of actions of different drugs. FA, fatty acid; sGC, soluble guanylate cyclase; SGLT2i, sodium-glucose cotransporter 2 inhibitor; RyR, Ryanod receptor; NHE-1, Na H^+^ exchanger; PGC-1α, peroxisome proliferator-activated receptor γ coactivator 1 α; Nrf2, nuclear erythroid 2-related factor 2; Ndufs1 NADH, ubiquinone oxidoreductase core subunit S1; mtCU, Ca^2+^ uniporter; CPT1, carnitine palmitoyl-transferase; SERCA2, sarcoplasmic–endoplasmic reticulum Ca^2+^ ATPase 2; SR, sarcoplasmic reticulum; A1AR, adenosine A1 receptor; mPTP, mitochondrial permeability transition pore; PLN, phospholamban; ROS, reactive oxygen species; MitoQ, mitoquinone.

Despite relevant decrease in cardiovascular (CV) mortality in the last years, the prognosis of HF patients is still poor. Based on strong evidences progressively derived from clinical studies on HF with reduced ejection fraction (HFrEF) (Figure 1), the most recent HF guidelines recommend the use of a foundational therapy including renin-angiotensin-aldosterone system inhibitors (RAASi), beta-blockers (BBs), mineralcorticoid receptor antagonists (MRAs) and sodium-glucose co-transporter 2 inhibitors (SGLT2i) to improve outcomes and reduce HF-related events (1). Recently, SGLT2i have also been shown to influence prognosis in HF patients with mildly reduced ejection fraction (HFmrEF) and preserved ejection fraction (HFpEF) (2, 3). Nonotheless, CV mortality remains considerable (4) in these patients, as new drugs are directed towards the usual and traditional pharmacological targets, not focusing the pathophysiologic “phenotypes”. HF patients belong to heterogeneous group, with different etiologies, comorbidities and so phenotypes. Clinical improvements derived from pharmacological therapy acting on neurohumoral and hemodynamic modulation may reach a plateau, with few possible additional benefits derived from additional therapies acting on the same pathway. The development of new drugs for HF must arise from new targets (as cardiomyocyte and myocardial interstitium, heart metabolism, GCs-cGMP pathway, mitochondrial function and intracellular calcium dysregulation) (5), identified through progressively better understanding of the complex pathophysiological mechanisms that occur in failing heart (6) (Figure 2). The increased knowledge of new metabolic pathways and cellular biology is crucial. Therefore, we describe main molecular pathways involved in HF pathophysiology, with adaptive and maladaptive effects on the CV system, associated with the current HF guidelines-recommended drugs, those with demonstrated benefits on HF outcomes but yet not included in guidelines, and new potential pharmacological targets for HF therapy (Table 1). In Tables 2, 3 we reported respectively already published and ongoing clinical trials that demonstrate the efficacy and safety of drugs acting on new targets in HF syndrome. Finally, a practical table (Table 4) with indications, contraindications, clinical end point and instrumental outcome and two summary (Figures 3, 4) figures were provided for each drug.

**Table 1:** Main molecular pathways involved in HF pathophysiology, therapeutic targets, second messengers, adaptive, and maladaptive effects on the cardiovascular system and related drugs.

Pathway	Receptor/target	Second messenger	Adaptive effects	Maladaptive effects	Drugs
Sympathetic nervous system	β1	Gs > AC > cAMP > PKA	Increased contractility and relaxation	Excitation contraction uncoupling Apoptotic pathways Alterations in β1 signals	*Selective β blockers (e.g., bisoprolol)*
	α1	Gq > PLC-β1 > DAG, IP3, PKC > MEF-2 ROS		Pro-fibrotic and pro-hypertrophic genes expression	*Non-selective β blockers (e.g., carvedilol)*
Renin–angiotensin–aldosterone system	AT-1	Gq > NADPH oxidase—ROS JAK/STAT > PTK PLC > DAG, IP3, PKC Tyrosine kinase—MAPK		Vasoconstriction, inflammation, proliferation, atherosclerosis Inflammation, growth, proliferation DAG, IP3 > vasoconstriction Inflammation, growth, proliferation	*ACE inhibitors, AT-1 antagonists (ARBs)*
	AT-2	Bradykinins > NO > cGMP Gq > PP2A, PTP > ↓ MAPK	Vasodilation, blunt in inflammation, growth and proliferation		*ACE inhibitors*
	MR	Tissues with 11-β-HSD2: Kidney: sodium-water retention vSMCs: galectin-3; PKB; PlGF Endothelium: ICAM. Tissues without 11-β-HSD2: Cardiomyocytes Macrophages: M1 phenotype	Increased contractility	Hypertension Fibrosis; apoptosis; atherosclerosis Leukocytes adhesion Hypertrophy, electric instability, oxidative stress Fibrosis and damage	*MR antagonists (MRAs)*
Natriuretic peptides	NPR-A	GC > cGMP > PKG	Vasodilation, diuresis, natriuresis, inhibition of cardiac hypertrophy and remodeling, suppression of ADH, blunt in SNS discharge		*Angiotensin receptor/Neprilysin inhibitor*
	NPR-B		Inhibition of vSMC proliferation, LDLox migration, ET-1 release		
	NPR-C	Internalization of NPs for degradation		Blunt in NPs effects	
Nitric oxide	Guanylate cyclase	cGMP	Vasodilation and muscular relaxation		*Soluble guanylate cyclase stimulators (vericiguat) and activators*
SGLT-2	SGLT-2	Glucose and sodium reabsorption		Glucose, sodium and water retention Activation of SNS? Oxidative stress? Cardiomyocyte metabolic impairment with usage of unfavorable substrates?	*Empagliflozin, Dapagliflozin*
Endothelin	ET-A	G_q_ > PLC > DAG, IP3 G_s_	Increased contractility	Fibrosis, arrhythmias, hypertrophy, vasoconstriction	*Endothelin receptor antagonists, both selective (-A or -B) or non-selective*
	ET-B	G_q_ > PLC > DAG, IP3 G_s_	cGMP-dependent endothelium-mediated vasodilation	Fibrosis, apoptosis, hypertrophy, PKC-dependent vasoconstriction	
Anti-diuretic hormone	V1a	G_q_ > PLC > DAG, IP3	Increased contractility	Vasoconstriction, platelets aggregation, hypertrophy	*Vaptans*
	V1b			Increased ACTH production	
	V2	G_s_ > AC > cAMP > aquaporin 2		Water reabsorption, hyponatremia	
Mitochondria	PPAR-α	Transactivation of genes for β-oxidation: CPT-1, MCAD		Induces shift to lipidic metabolism	*PPAR-α antagonists?*
	CPT			Stimulation of β-oxidation	*Trimetazidine*
	Cardiolipin	Mitochondrial membrane stabilizer	Efficient mitochondrial respiration		*Elamipretide?*
	Q10 coenzyme		Efficient mitochondrial respiration		*Q10 supplements*
Nox2 ROS	↓ PP-1 activity		Increased activity of SERCA and RyR	Impairment of the activity of both proteins, due to chronic oxidation	*Antioxidants?* *Elamipretide?*
Nox4 ROS	↓ PP-1 activity HIF stabilized		Angiogenesis	Hypertrophy	
Inflammation	e.g., IL-1				*Canakinumab?*
Fibrosis	TGF-β			Cardiac fibrosis	*Pirfenidone?*
	MMP				*MMP inhibitors?*
	Galectin-3				*Antisense RNA?*
TRPV-4	TGF-β_1_	Calcium	Vasodilation, arteriogenesis	Cardiac fibrosis, pulmonary hypertension, HF-related pulmonary edema	*TRPV-4 inhibitors?*

Taken from Ghionzoli et al. (5). AC, adenylate cyclase; cAMP, cyclic adenosine monophosphate; cGMP, cyclic guanosine monophosphate; CPT, acetyl-CoA C-acetyl transferase; DAG, diacyl-glycerol; ET, endothelin receptor; HIF, hypoxia inducible factor; HSD, hydroxysteroid dehydrogenase; ICAM, intercellular adhesion molecule 1; IL, interleukin; IP3, inositol triphosphate; JAK/STAT, Janus kinase/signal transducer and activator transcription factor; LDLox, oxidized LDL; MEF, myocyte enhancer factor; MMP, metalloproteinases; NADPH, nicotinamide-adenine dinucleotide; NO, nitric oxide; NP, natriuretic peptide; NPR, natriuretic peptide-binding receptor; PKA, protein kinase A; PKB, protein kinase B; PKC, protein kinase C; PKG, protein kinase G; PLC, phospholipase; PlGF, placental-derived growth factor; PP-, protein phosphatase-; PPAR, peroxisome proliferator-activated receptor; PTP, phosphotyrosine phosphatase; ROS, reactive oxygen species; RyR, ryanodine receptor; SERCA, sarco/endoplasmic reticulum calcium ATPase; TGF, transforming growth factor; TRPV, transient receptor potential cation channel subfamily V; vSMCs, vascular smooth muscle cells.

**Table 2:** Main evidences from clinical trials about novel HF targets.

Trial name	Metabolic modulation with perhexiline in chronic heart failure (7) (perhexiline vs. placebo) 2005	Vericiguat in patients with heart failure and reduced ejection fraction (VICTORIA) (8) (vericiguat vs. placebo) 2020	Effects of elamipretide on left ventricular function in patients with HFrEF (PROGRESS-HF) (9) (elamipretide vs. placebo) 2020	HORIZON-HF (10) (istaroxime vs. placebo) 2008	Cardiac myosin activation with omecamtiv mecarbil in systolic heart failure (GALACTIC-HF) (11) (omecamtiv mecarbil vs. placebo) 2021
Number of patients randomized	Perhexiline (*n* = 28) Placebo (*n* = 28)	Vericiguat (*n* = 2,526) Placebo (*n* = 2,524)	Elamipretide 4 mg (*n* = 23) Elamipretide 40 mg (*n* = 24) Placebo (*n* = 24)	Istaroxime (*n* = 89) Placebo (*n* = 31)	Omecamtiv mecarbil (*n* = 4,120) Placebo (*n* = 4,112)
Trial design	Double-blind, randomized, placebo-controlled study. Patients were allocated to ischemic (1) or nonischemic (2) group depending on coronary angiography. All patients underwent at baseline cardiopulmonary exercise testing, Minnesota Living with Heart Failure Questionnaire and 2D echocardiography. Group 1 underwent echo stress at baseline, Group 2 underwent MRS at baseline.	Multinational, randomized, double blind, placebo-controlled trial. After a screening phase of up to 30 days, eligible subjects were randomized in a 1:1 ratio to 2,5 mg of vericiguat or placebo. Then, based on SBP values, patient underwent uptitration to 5 mg and then 10 mg (target dose) of vericiguat.	Randomized, double-blinded, placebo-controlled, multiple dose study. Patients who met selection criteria were randomized in a 1:1:1 ratio, to receive either placebo, 4 mg elamipretide, or 40 mg elamipretide once daily by subcutaneous injection for 28 consecutive days. Total treatment duration was 4 weeks (visit 3), followed by a safety follow-up at 6 weeks after randomization.	Randomized, double-blind, placebo controlled, dose-escalation study. Patients were centrally randomized to istaroxime or placebo at a ratio of 3:1 within 3 sequential cohorts of 40 patients each. The first cohort: 0.5 μg/kg/min, The second: 1.0 μg/kg/min, The third: 1.5 μg/kg/min administered intravenously at a rate of 100 ml/h for 6 h.	International, multicenter, randomized, double-blind, placebo-controlled, event-driven CV outcomes trial. Patients were randomly assigned to receive either oral omecamtiv mecarbil or placebo in a 1:1 ratio. Postrandomization assessments were performed at weeks 2, 4, 6, 8, 12, 24, 36, and 48 and every 16 weeks thereafter.
Main inclusion criteria	LVEF <40% on echocardiography and optimally medicated HF with NYHA class II or III symptoms.	Chronic HF with NYHA class II, III, or IV and an LVEF < 45% and an elevated natriuretic peptide level within 30 days before randomization.	LVEF ≤ 40% with no hospitalization related to HF within 1 month prior to the screening visit and at least 3 dysfunctional but viable segments (hyperenhancement ≤ 25%) at cardiac MRI examination at screening.	LVEF ≤35%, hospitalized for HF with a SBP <150 and >90 mm Hg, heart rate <110 and >60 beats/min, and on standard HF therapy.	NYHA functional class II, III, or IV symptoms and a LVEF < 35%. Currently hospitalized for HF (inpatients) or been hospitalized for HF within 1 year before screening (outpatients).
Primary and main secondary endpoints	Peak exercise oxygen consumption (V˙O_2_ max) Effect of perhexiline on myocardial function (assessed with echo and MRS) and quality of life (assessed with Minnesota score)	Composite of death from cardiovascular causes or first hospitalization for HF. Components of the primary outcome, first and subsequent hospitalizations for HF, a composite of death from any cause or first hospitalization for HF, and death from any cause	Change from baseline in LVESV assessed by cardiac MRI Changes from baseline in LVEF and LVEDV by MRI. Echocardiographic end points (for ex. changes in E/A ratio, E/e’ ratio, biplane EF, left atrial volume, LV GLS). *Exploratory endpoints: changes in 6MWT, KCCQ score and NT-proBNP*	Change in PCWP compared with placebo after a 6-h continuous infusion. Changes in cardiac index CI, right atrial pressure, SBP, diastolic blood pressure, heart rate, and stroke work index.	A composite of a HF event or CV death, whichever occurred first, in a time-to-event analysis CV death, the change in the KCCQ score from baseline to week 24, the first HF hospitalization, and death from any cause
Median duration	8 weeks	10.8 months	6 weeks	6 h (continuous infusion of study medication)	21.8 months
Main results	Primary endpoint: significant improvements in V˙O_2_ max (16.1 ± 0.6 to 18.8 ± 1.1 ml kg^−1^ min^−1^; *P* < 0.001). Secondary endpoints: Improvement in quality of life (Minnesota score reduction from 45 ± 5 to 34 ± 5; *P* = 0.04) and left ventricular ejection fraction (24 ± 1% to 34 ± 2%; *P* < 0.001). No AE during the treatment period.	Primary endpoint: a primary-outcome event occurred in 897 of 2,526 patients (35.5%) in the vericiguat group and in 972 of 2,524 patients (38.5%) in the placebo group (HR, 0.90; 95% CI, 0.82 to 0.98; *P* = 0.02). Secondary endpoints: Slight reduction in hospitalizations for HF in the vericiguat group (HR, 0.90; 95% CI, 0.81 to 1.00) Slight reduction in CV deaths in the vericiguat group (HR, 0.93; 95% CI, 0.81 to 1.06) No statistical difference between the two groups for symptomatic hypotension or syncope.	Primary endpoint: nonsignificant differences from baseline to week 4 in LVESV between the three groups (4 mg vs. placebo: difference of means, −0.3; 95% CI, −4.6 to 4.0; *P* = 0.90; and 40 mg vs. placebo: difference of means, 2.3; 95% CI, −1.9 to 6.5; *P* = 0.28). Secondary and exploratory endpoints: No statistically significant increase in LVEF in either elamipretide 4 mg (*P* = 0.90) or 40 mg group compared with placebo (*P* = 0.37). Nonsignificant differences in 6MWT and NT-proBNP between the three groups. A trend towards improvement was observed in KCCQ score (*P* = 0.07). Rates of study drug related AEs were similar among the three groups.	Primary endpoint: All doses of istaroxime lowered PCWP (mean ± SD: −3.2 ± 6.8 mm Hg, −3.3 ± 5.5 mm Hg, and −4.7 ± 5.9 mm Hg compared with 0.0 ± 3.6 mm Hg with placebo; *P* < 0.05 for all doses). Secondary endpoints: Increase in SBP at the end of the 6-h infusion (*P* = 0.02 and <0.001, respectively). Decrease in heart rate in a dose-dependent manner (*P* = 0.008, 0.02, and 0.006, with 0.5, 1.0 and 1.5 μg/kg/min, respectively) Increase of cardiac index during the 1.5 μg/kg/min infusion (*P* = 0.04 vs. placebo) AEs were not life threatening and were dose related.	Primary endpoint: a primary-outcome event occurred in 1,523 of 4,120 patients (37.0%) in the omecamtiv mecarbil group and in 1,607 of 4,112 patients (39.1%) in the placebo group (HR, 0.92; 95% CI, 0.86 to 0.99; *P* = 0.03). Secondary endpoints: no significant difference between groups for CV death (HR, 1.01; 95% CI, 0.92 to 1.11; *P* = 0.86), first hospitalization for HF (, 0.95; 95% CI, 0.87 to 1.03) and all-cause death (HR, 1.00; 95% CI, 0.92 to 1.09). The frequency of cardiac ischemic and ventricular arrhythmia events was similar in the two groups.

MRS, ^31^P magnetic resonance spectroscopy; LVEF, left ventricular ejection fraction; NYHA, New York Heart Association; CI, confidence interval; HR, hazard ratio; AE, adverse event; MRI, magnetic resonance imaging; LVESV, left ventricular end systolic volume; LVEDV, left ventricular end diastolic volume; GLS, global longitudinal strain; 6MWT, 6-minutes walking test; KCCQ, Kansas City cardiomyopathy questionnaire; SBP, systolic blood pressure; DBP, diastolic blood pressure; PCWP, pulmonary capillary wedge pressure.

**Table 3:** Ongoing clinical trials about novel HF targets.

Trial name	Evaluation of the cardiac and metabolic effects of semaglutide in heart failure with preserved ejection fraction (CAMEO-SEMA) (12) (semaglutide vs. placebo)	Examining the effects of mitochondrial oxidative stress in dilated cardiomyopathy (13) (mitoquinol mesylate vs. placebo)	Modulation of SERCA2a of intra-myocytic calcium trafficking in heart failure with reduced ejection fraction (MUSIC-HFrEF1) (14) (SRD-001 AAV1/SERCA2a vs. placebo)	A Study of vericiguat (MK-1242) in participants with chronic heart failure with reduced ejection fraction (HFrEF) (MK-1242-035) (VICTOR) (15) (vericiguat vs. placebo)	Exploratory study of danicamtiv in patients with primary dilated cardiomyopathy (DCM) due to genetic variants (16)
Estimated enrollment	81 participants	106 participants	56 participants	6,000 participants	24 participants
Trial design and aim of the study	A Phase II, Prospective, Double-Blind Randomized Trial. Patients will receive the study medication once weekly in addition to counselling on healthy lifestyle intervention (behavioral intervention). The purpose of this research is to find out if an aggressive intervention to lose weight, will improve symptoms in patients with obesity-related cardiomyopathy, which is also known as the obese phenotype of HFpEF.	A Phase II, double blind, randomised, placebo-controlled trial of MitoQ (mitoquinol mesylate) in patients with dilated cardiomyopathy, examining the effect of reducing mitochondrial oxidative stress on myocardial energetics and myocardial function using 31-phosphorus magnetic resonance spectroscopy and cardiovascular magnetic resonance.	A Phase 1/2, Prospective, Double-Blind, Randomized Trial of the Safety and Efficacy of SRD-001 an adeno-associated virus serotype 1 (AAV1) vector expressing the transgene for sarco(endo)plasmic reticulum Ca^2+^ ATPase 2a isoform (SERCA2a), in anti-AAV1 neutralizing antibody (NAb) negative subjects with ischemic or non-ischemic cardiomyopathy and New York Heart Association (NYHA) class III/IV symptoms of heart failure with reduced ejection fraction (HFrEF)	A Pivotal Phase 3 Randomized, Placebo-controlled Clinical Study to Evaluate the Efficacy and Safety of the sGC Stimulator Vericiguat/MK-1,242 in Adults With Chronic HFrEF.	A phase 2a study aiming at establishing safety and preliminary efficacy of danicamtiv in patients with primary dilated cardiomyopathy (DCM) due to MYH7 or TTN variants.
Main inclusion criteria	BMI ≥ 30.0 kg/m^2^, NYHA Class II–IV, LVEF ≥ 50% within the preceding year, no hospitalizations due to HF in the preceding 30 days, high filling pressures or pulmonary hypertension, echo signs of structural heart disease, and elevated NT-proBNP levels	Idiopathic or familial DCM LVEF ≤45% on 2 imaging studies. On guideline therapy for ≥3 months. Sinus rhythm on 12-lead electrocardiogram. Plasma NT-pro-BNP >250 ng/L for those >65 years and >100 ng/L for those aged ≤65 years within the last 6 months	Chronic ischemic or non-ischemic cardiomyopathy; NYHA class III/IV; LVEF ≤35%; Maximal, optimized heart failure therapy; ICD.	History of chronic HF (NYHA) Class II to IV on guideline-directed medical therapy for heart failure with no HF hospitalization within 6 months or outpatient IV diuretic use within 3 months before randomization. LVEF ≤ 40%. Elevated NT-proBNP levels.	Stable primary DCM due to MYH7 or TTN variants; good echocardiographic acoustic windows; up to 3 family members with the same variant can be enrolled
Endpoints	Change in PCWP during exercise from baseline after 12 months of treatment	Change in LVESVi (ml/m^2^) between baseline and follow-up amongst all 106 patients measured using cardiovascular magnetic resonance	Change in NYHA class, Kansas City Cardiomyopathy Questionnaire, 6MWT, LVESV, recurrent events and adverse events from baseline after 6 and 12 months of treatment	Time to First Occurrence of Composite Endpoint of Cardiovascular (CV) Death or Heart Failure (HF) Hospitalization	Frequency and severity of treatment-emergent adverse events and serious adverse events.
Estimated completion date	August 2026	July 2025	December 2028	June 2025	January 2025

BMI, body mass index; LVEF, left ventricular ejection fraction; NYHA, New York Heart Association; PCWP, pulmonary capillary wedge pressure.

**Table 4:** Indications, contraindications, clinical end-point and strumental outcome for each drug.

Drug	Indications	Contraindications	Impact on clinical end point
**Drugs targeting cardiac metabolism**			
Carnitine palmitoyltransferase I (CPT1) inhibitors (perhexiline and etomoxir)	- Chronic HF - Hypertrophic cardiomyopathy - Dilated cardiomyopathy	Etomoxir: - liver function test impairment - peripheral neuropathy	Improvement: - VO_2_ max - LVEF - Symptoms - Resting and peak stress myocardial function
Inhibitor of 3 ketoacyl-CoA thiolase (Trimetazidine)	- Ischemic cardiomyopathy - Diabetic pat. with HF	Parkinson symptoms - restless leg syndrome - tremors and gait instability - severe renal failure	Improvement: - LVEF - NT-proBNP levels Reduces all-cause mortality and HF hospitalization
Pyruvate dehydrogenase kinase (PDK) inhibitor dichloroacetate (DCA)		- neurotoxicity	Improvement: - LV function - Exercise capacity
Glucagon-like peptide-1 receptor agonists (GLP-1RAs)	- HFpEF patients with obese phenotype - HF patients with high-risk for or with atherosclerotic cardiovascular disease (ASCVD)	- Severe gastrointestinal diseases (gastroparesis and inflammatory bowel) - Personal or family history significant for multiple endocrine neoplasia 2A, multiple endocrine neoplasia 2B, or medullary thyroid cancer - Pancreatitis - Severe renal dysfunction	Improvement: MACE Neutral effects on hospitalization for HF and mortality
**Drugs acting on NO-sGC pathway**			
Soluble Guanylate Cyclase (sGC) Stimulator (Vericiguat)	CHF patients (LVEF <45%, decompensation within 6 months and elevated BNP)	- Coadministration with PDE-5 inhibitors - GFR <15 ml/min/1.73 m^2^ - Child pugh C	reduction of the composite end-point of death from any cause or hospitalization for HF
**Drugs acting on mitochondrial function**			
Adenosin receptor agonist (Capadenoson, Neladenoson)	advanced HF		Improvement: - cardiac fibrosis - cardiac remodeling
Elamipretide	HFrEF		Improvement: - LVEF - LV remodeling
Mitoquinone (MitoQ)	- Hypertrophic cardiomyopathy - Dilated cardiomyopathy		
Mitochondria-localized circular RNAs (circRNAs)			Improvement: cardiac remodelling
**Drugs acting on intracellular calcium dysregulation**			
Istaroxime	AHF		Improvement: - risk of arrhythmias - Cardiac index - Pulmonary capillary wedge pressure - Systolic blood pressure
SERCA2a gene therapy	CHF		Improvement: - symptoms - NT-proBNP levels - risk of recurrent CV events
Ryanodine receptor channel (JTV519 (K201)	Ischemic cardiomyopathy		Improvement: - cardiac contractility - cardiac dysfunction - -ventricular remodelling
Cimlanod (nitroxyl donor)	AHF and CHF with HFrEF		Improvement: - cardiac index - diastolic function
NHE-1 inhibitors (cariporide, eniporide, zoniporide)	- Hypertrophic cardiomyopathy - Ischemic cardiomyopathy	ischemic stroke	Improvement: - LV end-systolic volume - LVEF - arrhythmias
**Drugs acting on cardiac myosin**			
Cardiac myosin activator (Omecamtiv Mecarbil, Danicamtiv)	CHF (NYHA FC II-IV), LVEF ≤35%, inpatients or urgent ED visit or HF hospitalization within 12 months), Elevated BNP/NT-proBNP	SBP <85 mmHg - GFR <20 ml/min/1.73 m^2^	Improvement: time to first HF event or CV death - systolic ejection time - stroke volume - LV diameters and volumes

HF, heart failure; AHF, acute heart failure; CHF, chonic heart failure; HFrEF, heart failure reduce ejection fraction; LV, left ventricle; CV, cardiovascular; LVEF, left ventricular ejection fraction; NYHA, New York Heart Association; BNP/NT-proBNP, brain natriuretic peptide/N-terminal-pro brain natriuretic peptide; eGFR, estimated *glomerular filtration rate*; SBP, systolic blood pressure.

## 2. Drugs targeting cardiac metabolism

The heart needs high energy and can exploit many substrates to maintain its proper function. In fact, it produces and consumes approximatively 6 kg of ATP every day and has high oxygen demand to maintain its work. It possesses an efficient and complex machinery capable of producing ATP starting from various substrates, such as fatty acid (FA), carbohydrate, ketone bodies (KB), and, less frequenly, pyruvate, lactate, and aminoacids (17). FA is the most important fuel source for the heart (18), producing energy by FA β-oxidation (FAO) (19). Interestingly, FAO consumes 11%–12% more oxygen for producing the same quantity of ATP in comparison with glucose, proving to be a less efficient substrate (17). Glucose involved in cardiac metabolism derives from the usage of exogenous glucose or glycogen stores. Despite its crucial role in supporting cardiac contractile function is attested by several evidences (20), a recent study highlighted that heart under physiological conditions makes use of a smaller quantity of glucose compared to the expected one (21). Regarding KB, their circulating levels under normal physiological conditions are low. However, starvation, ketogenic diet, and strenuous physical activity can increase ketone levels (17). KBs are able to develop more energy per 2 carbons if compared with glucose, proving to be an effective substrate, despite they are overall energetically less efficient than glucose. Nevertheless, the cardiac muscle rapidly provides for their oxidation and an up-to-date mapping of fuel uptake in human heart showed that under normal physiological conditions it also uses a significant amount of KB (17, 21).

Impaired cardiac metabolism in HF patients with HFrEF has been widely studied, while metabolic changes in HFpEF remain extensively not known (17). It is widely accepted that HF is associated with cardiac metabolic alterations at various levels, leading to decrease in ATP production (22). In fact, clinical trials showed that the failing myocardium undergoes a 30% reduction in ATP levels, in comparison with healthy myocardium, associated with a reduced (ATP?)flux via creatine kinase (23). Moreover, the reduction in ATP production seems to be related to changes in the myocardium, with a shift from FA towards increased glucose utilization. In particular, HF results in inadequate oxygen delivery to the myocardium and disposal of metabolic wastes, with the result that cardiomyocytes become less efficient in using FAs as energy sources. The aforementioned metabolic switch in an indirect way induces glycolysis and at the same time determines a decrease in glucose oxidation (17). This phenomenon was noted in animals with HF, pigs with pacing-induced HF and humans affected by end-stage HF (24). Although evidences seem to suggest a reduced FA oxidation and a decoupling among glycolysis and glucose oxidation in HF, also opposing data have been discovered: iy was registered an increased glucose oxidation in dogs with pacing-induced HF (25) and a major recourse to glucose oxidation were among patients with idiopathic dilated cardiomyopathy (DCM) (26). Therefore, the different metabolic unbalance in the heart may vary on the basis of the experimental model (in experimental studies), kind and gravity of cardiac dysfunction, and in response to distinct pathological stimuli (17). Interestingly, as HF progresses, there is also a reduction in glucose oxidation, increasing the relevance of KB as an energy resource for cardiac metabolism (27). In fact, it seems that KBs can act as an alternative energetic substrate for the failing heart. As a proof of this, an increase in circulating KBs and in ketone oxidation rates were registered in HFrEF and HFpEF (27) and their oxidation seems to represent around the 20% of the total produced cardiac energy in small animal models of HFrEF (28), similarly to data observed in patients with HFrEF (21). Although few studies have directly assessed KB metabolism in myocardium, it has been observed a strong correlation between circulating ketone levels and myocardial ketone oxidation (17, 21). On the whole, the existing evidences support the hypothesis of an high recourse to KB oxidation in the failing heart as an energy resource (17).

Since accumulating evidences shows a relation between imbalanced cardiac metabolism and HF development and progression, these altered energetic pathways offer themselves as appealing therapeutic targets. Different drugs that display, directly and indirectly, a modulation on myocardial metabolism substrates, have been created and tested in HF patients. The final purpose is to ensure the best possible energy efficiency, both allowing better inotropy and lusitropy and averting more energy expenditure. There are five main goals that can be pursued in therapy targeting cardiac metabolism in HF:

1. Inhibition of FA uptake by cardiomyocytes
2. Reduction of FA oxidation
3. Reduction of Circulating FA Levels
4. Increase of Glucose Oxidation
5. Increase of Ketone Oxidation

During the pathophysiological progression of HF, there is a reduction in FA and glucose oxidation determining, through an adaptive mechanism, an increase in ketone metabolism. Recent evidences suggest a beneficial effect of therapeutic ketosis, in particular in HFrEF, with also a theoretical rationale for its use in HFpEF (29). About the inhibition of FA uptake, Carnitine palmitoyl-transferase (CPT1) is the limiting enzyme for FA oxidation, because regulates FA entrance into mitochondria and thereby can be a potential drug target,. Administration of etomoxir and perhexiline, two CPT1 inhibitors, results in reduced FA oxidation and boosted glucose oxidation. In a small pilot study it has been demonstrated that etomoxir can improve cardiac performance in HF patients (30). Perhexiline, initially used to treat angina pectoris, is a specific cardiac isoform of CPT1 inhibitor. When studied, perhexiline was able to improve VO_2_ max, left ventricular ejection fraction, and myocardial energetics in chronic HF (7). Moreover, patients with hypertrophic cardiomyopathy treated with perhexiline showed a better exercise capacity and myocardial energetics (17). In patients with DCM, perhexiline increased phosphocreatine to adenosine triphosphate (PCr/ATP) ratio and NYHA functional class, without any change in LVEF or cardiac substrate usage (31). On the contrary, in hypertrophy linked to aortic stenosis (AS), perhexiline didn't show benefits in ameliorating hemodynamic performance or on the degree of myocardial injury (32). The reasons why CPT-1/2 inhibitors have different effects on different types of HF are not completely known. For example, perhexiline is a drug currently used to treat refractory angina pectoris, which appears to act primarily as a potent inhibitor of carnitine palmitoyl transferase-1 (CPT-1) by hindering the transport of long-chain FAs into the mitochondria and thereby shifting myocardial metabolism toward more energy-efficient glucose utilization, potentially correcting the myocardial energy deficit. It appears to provide clinical benefits in HF, regardless of ischemic or nonischemic etiology, as evidenced by some clinical studies (7).

There are some energetic and metabolic theories that partially explain why these drugs show clinical benefits in some forms of HF. For example in hypertrophic cardiomyopathy (HCM), according to the “energy depletion hypothesis”, it has been suggested that impaired myocardial energetics could have a role in the development of left ventricular hypertrophy (LVH). In particular, biophysical studies of the different classes of HCM mutant proteins have shown a reduced mechanical efficiency with consequent increase in the energy cost of force production. Moreover, genotype positive and phenotype negative HCM patient's shows an impairment of myocardial energetics. Furthermore, the previously published data from Crilley et al. hypothesize that the development of the hypertrophic phenotype in HCM occurs as a secondary response to derangement of myocardial metabolism (33). It has been hypothesize that perhexiline may improve myocardial energetics, limiting the occurrence of myocardial ischaemia and progressively reversing LVH in HCM. In particular, the suppression of FA oxidation, by cross-talk between cycle-specific inhibitors, may improve myocardial energy efficiency shifting myocardial substrate utilization toward glucose metabolism, known as a “Randle Shift”. Secondary induction of glucose utilization leads to reverse impaired myocardial energetics. Perhexiline enhances effects of nitric oxide potentially improving microvascular function, and exert an anti–inflammatory action independent of CPT inhibition. Evidence suggests that perhexiline may improve symptomatology in HCM patients. The ongoing RESOLVE-HCM trial will clarify if perhexiline may revert LVH in symptomatic HCM patients (34). On the other hand, HYPER trial assessed the role of perhexiline as a metabolic modulator aiming to confer myocardial protection in patients with LVH secondary to AS undergoing surgical aortic valve replacement (SAVR) (32). The hypothesis was that energetically compromised hypertrophied myocardium might have been more vulnerable to ischemia/reperfusion injury and thus might have benefited more from improved metabolism. HYPER trial was the first to have assessed the role of perhexiline in this kind of patients. Unfortunately perhexiline therapy as an adjunct to standard myocardial protection resulted in no overall benefit in patients undergoing SAVR ± Coronary Artery Bypass Graft. This trial had some important limitations, that could partially explain these results. First of all, it was a nonrandomized trial with a small number of participants. Furthermore, there were no patients who did not undergo cardiac surgery even though considered eligible for surgery. In addition, all the studies that support perhexiline as a metabolic agent share the common characteristic that therapy is prolonged, monitored and optimized over months. This is not pragmatically possible in the real-world practice of cardiac surgery, reflected by this study. Another important point is that 39% of patients were below the therapeutic range of serum perhexiline concentration. Further demonstrating that these limitations may have weighted on the final result, there have been other studies advocating perhexiline as a clinically useful metabolic therapy in AS. For example, a small study evaluated the use of perhexiline in elderly symptomatic AS patients, showing a great improvement in clinical picture of these patients (35). However, this study had the important limitations of the small number of participants and its non-randomized nature. So the use of perhexiline as a modulator of myocardial metabolism may remain limited to patients who are not candidates to cardiac surgery and refractory to an optimized medical therapy. Importantly, potential neurotoxic and hepatotoxic adverse effects have been highlighted with the use of CPT1 inhibitors (perhexiline and etomoxir), making their clinical use limited (17).

β-oxidation of FAs involves mitochondrial enzymes that may become a target in patients with HF, aiming to reduce FA oxidation. Trimetazidine (TMZ) plays a competitive inhibition of 3-ketoacyl coenzyme A thiolase, the ultimate enzyme of FA oxidation and has been administered in clinical practice for stable coronary artery disease (17). In recent years it has also emerged as a novel option for the treatment of HF (36). Although its mechanism of action is not fully understood, TMZ has shown to decrease free FA oxidation, inhibit oxidative phosphorylation, improve glucose utilization and ATP production. In addition, TMZ seems to have other beneficial effects, as conservation in cardiomyocytes of ATP and phosphocreatine content, reduction of cell acidosis, calcium overload and mitigation of cell injury caused by oxidative stress. In the past, small randomized trials evaluated the therapeutic role of TMZ in patients affected by HF and ischaemic etiology with promising results, including improvement in symptoms and ejection fraction and reducing hospitalization rates (37). In particular, administration of TMZ has been associated with improvements in functional class (38) and LVEF (38–40) in HF patients. A similar positive effect on LVEF has been reported in patients with idiopathic DCM (41) and diabetic cardiomyopathy (42). Evidences suggest that the improvement of LVEF is more prominent when trimetazidine is used with β-blockers, assuming a synergistic effect (41). Conversely, in a more recent study no benefit on cardiac function, functional capacity and quality of life was observed with trimetazidine added to standard medical therapy in patients with stable nonischemic HF (43). There are possible explanations for these discordant results: first of all, the beneficial effects of TMZ on symptoms and ejection fraction in patients with HF caused by coronary disease may partially be explained by its antianginal effect. In fact, two recent meta-analyses of studies conducted in patients with HF, most of them with ischaemic etiology, revealed a reduction in LV end-diastolic diameter with an improvement of left ventricular ejection fraction (about 7%) (44). On the other hand, in non-ischemic HF evidences about the therapeutic role of TMZ are poor. In a small study conducted by Gunes et al. 87 patients with both ischemic (69% of patients) and non-ischemic HF and LVEF ≤40% were treated with TMZ (*n* = 51) or placebo (*n* = 36). After 3 months, there was an improvements in LVEF and tissue Doppler velocities, but patients with both ischemic HF and diabetes mellitus showed the greater improvement in LVEF (45). Another possible explanation is that TMZ may be more relevant in patients with impaired oxygen supply, as for example in coronary disease, because of its action on myocardial metabolism with a shift from FFA to glucose utilization as the primary myocardial energetic substrate. Furthermore, TMZ showed more beneficial effects in patients with metabolic disorders associated with increased FFA oxidation, such as in obesity and diabetes mellitus. In the cohort of the study we cited, there was a small prevalence of diabetic patients (8%) and the mean body mass index was discretely over normal limits. These aspects could partially explain the lack of benefit of TMZ treatment in stable non-ischemic HF patients, so despite of its apparent good safety profile in these patients, the lack of beneficial effects do not yet support the use of TMZ in this cohort.

A valid approach to indirectly balance FA oxidation in HF could be to reduce circulating levels of FA. Among others, β-blockers (metoprolol and carvedilol) showed to improve cardiac performance and survival in patients with HF (46–48), because of their ability of sparing energy, decreasing myocardial FA use (48) and augmenting carbohydrate oxidation (49). Moreover, metoprolol exerts the same benefit of carvedilol with no reduction of free FA (49). Niacin could decrease myocardial FA oxidation by decreasing circulating FA levels. However, studies about the nicotinic acid derivative acipimox in DCM (50) and ischemic HF (51) showed both a significantly reduced cardiac function and efficiency and a significant decline in circulating FA. Furthermore, to reduce FA delivery to the cardiac muscle through peroxisome proliferator–activated receptor (PPAR) α and PPAR γ agonists (fibrates and thiazolidinediones) did not avoid the onset of HF. Treatment with thiazolidinedione has been paired to the risk of new or aggravated HF, too (52, 53).

Pyruvate dehydrogenase kinase (PDK) inhibitor dichloroacetate (DCA) showed to be beneficial in preclinical models of HF (54). DCA, that is an analog of pyruvate, leads to an increase in PDK function and thus facilitates glucose oxidation. Though, DCA demonstrated no benefit on LV performance or exercise capacity in HF patients (55). Moreover, DCA's chronic neurotoxicity limits its possible evaluation in human studies (56). Glucagon-like peptide-1 receptor agonists (GLP-1RAs) are recommended in all patients with T2DM who are at high CV risk or have an atherosclerotic CV disease (ASCVD) yet (57, 58). The postulation is that GLP1-RAs may prevent from HF because they cause an increased glucose uptake and usage (17). Preclinical studies supported this hypothesis, showing that GLP-1RAs augment myocardial glucose uptake (59, 60).

GLP1-RAs, through increasing glucose uptake and its utilization by myocardial cells, may prevent the development of HF. A lot of experimental data about the role of GLP-1 in diabetes has been produced, but there are very limited evidences about its CV molecular effects (61). The CV effects attributable to GLP1-RAs can be summarized as:

- Counteracting inflammation, oxidative stress and atherosclerosis: preclinical studies showed that GLP-1RAs may directly prevent from atherogenesis modulating vascular inflammation by suppressing the migration and accumulation of monocytes and macrophages into the arterial wall, thanks to a downregulation of different inflammatory and adhesion molecules on the aforementioned cells. It has been shown that, in some studies, liraglutide could retard the progression of atherosclerotic plaque formation and stabilize the plaque. Liraglutide may also reduce TNF-α-induced reactive oxygen species (ROS) production and inflammation in endothelial cells. In particular, it seems that liraglutide could suppress endothelial cell inflammation through a calcium- and AMPK-dependent mechanism. Moreover GLP-1RAs may minimize innate immune response in type II diabetes mellitus patients reducing the pro-inflammatory cytokines (TNF-α, IL-1β, IL-6), and increasing the production of adiponectin (that is an adipokine with anti-inflammatory effects). Additionally, GLP-1-RAs could abolish the macrophage foam cell formation carried out by oxidized LDL in a receptor-dependent way, linked to an increased activity of acyl-coenzyme A cholesterol acyltransferase-1 and CD36, and a decreased one of ATP-binding cassette transporter A1.
- Reducing Oxidative Stress-Induced Endothelial Dysfunction and counteracting thrombosis: Oeseburg et al. demonstrated that GLP-1RAs are able to avoid ROS inducing the expression of antioxidant genes (as NQO1 and HO-1) after the activation of the protein kinase A (PKA) and cAMP response element-binding (CREB) protein. Moreover, GLP1-RAs seems to attenuate upregulation of macrophage scavenger receptors CD36 and lectin-like oxidized low-density lipoprotein scavenger receptor-1 (Lox-1), providing protection against vascular injuries. Moreover, growing evidence shows that GLP1-RAs can inhibit thrombus growth formation via eNOS activation.
- Diuretic and natriuretic effect: GLP-1R appears in the renal proximal tubular brush border too, where it might control Na^+^ reabsorption. It seems that GLP1-RAs could increase the expression of phosphorylated Na^+^/H^+^ exchanger NHE3 on the kidneys membrane, resulting in increased eGFR, fractional Na^+^ excretion and urinary excretion.
- Renin-angiotensin-aldosterone effect: preclinical studies on rat models of type-1 diabetes showed that liraglutide may revert the imbalance in the renin angiotensin system, reversing RV hypertrophy too. Moreover, GLP1-RAs seem to increase lung ACE2 expression and circulating Angiotensin (1–7), a process that stimulates systemic vasodilatation (61).

Nevertheless, human studies do not provide sufficient evidence to support the hypothesis that this mechanism induced by GLP-1RAs therapy mediates cardioprotection. For example, metabolic parameters in patients with congestive HF and without diabetes were not changed by a 48-h subcutaneous administration of GLP-1 (62). Besides, GLP1 enhanced myocardial blood flow with no changes in myocardial glucose uptake in patients with diabetes and without coronary artery disease (63). A clinical trial is currently underway to evaluate the cardiac and metabolic effects of semaglutide, a new-generation GLP1RA, in HFpEF patients with obese phenotype (12).

Another reasonable effective manner to supply to the heart a more energy-efficient substrate, useful strategy in HF, is by increasing ketone oxidation (17). Ketone might exert extra pleiotropic actions in addiotion to the cardiac energetic ones. Ketone oxidation rate and its arterial concentration are directly proportional; thus, if there is an increased ketone supply to the heart, this will result in increased cardiac ketone utilization (64). Increased ketone levels are reached with fasting, ketogenic diet, medium-chain triglyceride (MCT), 1,3-butanediol, ketone salts (KSs), or ketone ester (KE) supplementation. Remarkably, it has also been reported that SGLT2i can augment circulating ketone levels and cardiac energetics, and this might apport clinical advantages to the failing heart (65–67). Moreover, SGLT2i administration may modulate the nucleotide-binding oligomerization domain (NOD)-like receptor protein 3 (NLRP3) inflammasome through increasing circulating ketones, thereby reducing inflammation in the failing heart (68). Preclinical models of HF had previously shown a significantly favourable effect of fasting, ketogenic diet, medium-chain triglyceride (MCT), ketone supplementation and higher ketone levels reached through SGLT2i utilization (17). For example, supplementing with KE mitigated LV dysfunction and remodeling in two preclinical models of HFrEF (69). Benefits of ketone supplementing in HFpEF are not completely understood. In a mice model with HFpEF (based on age, long-term high-fat diet, and deoxycorticosterone pivalate challenge), higher circulating ketones levels proved to be beneficial through the suppression of proinflammatory cytokines, an improved mitochondrial function, and the mitigation of cardiac fibrosis, despite reduced ketone oxidation (70). Conversely, opposite results were reported in non-HF animals (17). In fact, long-term ketogenic diet induced cardiac fibrosis and reduced cardiac function in healthy adult mices (71). Up to now, researchers only tested ketone salts and ketone esters in HF (72, 73). A 3 h-infusion of KSs Sodium-β-hydroxybutyrate (Na-βOHB) boosted cardiac output and lowered systemic vascular resistance in HFrEF patients (72), while an acute oral supplementation with KE promoted ketosis and increased cardiac ketone usage, and it was associated with the degree of cardiac function and remodeling in HFrEF patients (73).

In conclusion, in recent years, equally experimental and clinical studies gave numerous insights into cardiac metabolism, major myocardial metabolic substrates, and their pathophysiological alterations in particular in HF. Based on this evidence, different metabolic compounds have been developed and tried out over time with the aim of ensuring improved myocardial metabolism and energetics. Although these studies have surely provided an expansion of knowledge in this area, the evidence is currently insufficient to recommend a “metabolic therapy” in HF. Further research, particularly in rigorous clinical trials conducted in large populations, is therefore required to study safety, feasibility, and efficacy of “metabolic” therapy in HF.

## 3. Drugs acting on NO-sGC pathway

In HF, nitric oxide (NO) production is greatly decreased due to several mechanisms, that include inhibition of endothelial NO synthase and deactivation by ROS, particularly superoxide dismutase (74). All this leads to vasoconstriction and then to raised cardiac preload and afterload, increased muscular and vascular stiffness and unfavorable remodeling (1, 75). In the myocardium, NO mitigates the action of the calcium channel, sarcoplasmic reticulum calcium adenosine triphosphatase (SERCA) pump, sarcoplasmic reticulum and ryanodine receptor, with an impact on mitochondrial metabolism (76). Therefore, restoring NO-sGC-cGMP (cyclic guanosine monophosphate) pathway plays a crucial role to relieve the HF burden (75, 77).

Many compounds that targets the NO-GC signaling have been elaborated. There are three classes of drugs with different action: some drugs directly operate on the soluble form of GC and are categorized as activators (cinaciguat) and stimulators (vericiguat and riociguat) (78), others decrease cGMP degradation, the Phosphodiesterase 5 inhibitors (such as sildenafil) (79). Cinaciguat activates the sGC by mimicking NO itself. In patients with HF, cinaciguat decreased the pulmonary capillary wedge pressure (PCWP), but also was associated to the increase of low blood pressure, which led to early withdrawal from this trial (80). On the other hand, GC stimulators enhance soluble CG activity when the endogenous ligand is available (77).

Vericiguat, a new soluble GC (sGC) stimulator, acts as a stimulator of sGC, *playing* synergistically with available NO, leading to an increase in cGMP levels with *improvement of* cardiac and vascular function (81).

Vericiguat was tested in phase II SOCRATES PRESERVED (82) and REDUCED studies (83) and recently also in the phase III VICTORIA study, with reduction of the composite end-point of death from any cause or hospitalization for HF versus placebo in subjects with worsening HFrEF (8). Therefore, this medication is also recommended in the existing HF guidelines (class II, level of evidence B) (2). Vericiguat, compared with the other drugs of this class such as riociguat, which did not reach the primary endpoint in phase II LEPHT (Riociguat in Patients with Pulmonary Hypertension Associated with Left Ventricular Systolic Dysfunction) study (84), induces a minor reduction in blood pressure, thanks to these characteristics: increased pharmacokinetic stability, higher oral bioavailability and an extended half-life (85).

In the VITALITY-HFpEF trial, the vericiguat has not increased the quality of life evaluated by the Kansas City Cardiomyopathy Questionnaire (KCCQ) score in HFpEF patients (86). This may be consistent with the assumption that NO deficiency has no key role in developing HFpEF, as opposed to HFrEF.

Patients with a recent worsening HF event and a baseline NT-proBNP value ≥8,000 pg/ml are the ones who benefit the most from this drug (87). These encouraging results may bring the use of a quintuple treatment through the introduction of vericiguat as a new drug for the treatment of HFrEF, along with ACEi/ARB/ARNI, beta-blockers, MRA, and SGLTi (88). A clinical trial evaluating the impact of vericiguat on outcomes (CV mortality and HF hospitalization) in patients with stable chronic HFrEF is ongoing (15).

## 4. Drugs acting on mitochondrial function

Mitochondria play an important role in the cardiac pathophysiology by affecting cardiac function and metabolism. The finding of fragmented and damaged mitochondria are a hallmark of heart disease. Dysfunction of systems preserving mitochondrial anatomy (number, size, and shape) through fission/fusion and mitophagy affects mitochondrial function (89), breaks up cellular bioenergetics, and produces increase of oxidative stress. Impaired mitochondrial function leads to calcium dysregulation and, above all, cardiomyocyte death.

In HF models, increase of ROS, the prolonged opening of the mitochondrial permeability transition pore (mPTP) and aberrant mitochondrial dysfunction (90) are harmful to mitochondrial metabolism and negatively affect cardiac structure and function (91). mPTP opening is a mitochondrial response to an oxidative challenge resulting in an increased ROS signal. The ROS thus derived, may be drivers of cardiomyocyte (CM) cell-cycle arrest.

Drug development research for HF is also focused on mitochondria, involved in the production of high-energy molecules and initiation of programmed cell death. They are ideal therapeutic targets because placed between metabolic and energetic pathways affecting cardiac function (92).

There are new results justifying the use of drugs targeting mitochondrial fission and fusion in different CV diseases (93). The present challenge is to transform these new molecules into drugs. Peroxisome proliferator-activated receptor γ coactivator 1 α (PGC-1α) acts as a transcriptional cofactor, by modulating mitochondrial biogenesis and mitochondrial dynamics and mitophagy. Impaired regulation of PGC-1α function is correlated to the onset and progression of HF (94). Nuclear erythroid 2-related factor 2 (Nrf2), a transcription factor of endogenous antioxidant defense systems against oxidative stress (OS), *may be* a *potential* therapeutic target for management of some CV diseases (as HF). Natural products may be a *possible* source of Nrf2 activators with cardioprotective activities *mediated* by suppression of OS providing a novel therapeutic target for HF (95).

Adenosine A_1_ receptors (A_1_AR) are a possible new target for new therapies thanks to their cardioprotective/antihypertrophic properties (96). Stimulation of adenosine receptor (AR) seems to lead a cytoprotective action against oxidative injury. Capadenoson, classified as an A_1_AR partial agonist, was demonstrated to improve LV remodeling in advanced HF. The therapeutic effects of capadenoson may be mediated through the A_1_AR. Capadenoson have significant A_2B_AR activity in cardiomyocytes. Baltos et al. (97) suggested a development of capadenoson-like molecules, as the A_2B_AR able to promote cardioprotection and reduce cardiac fibrosis in CV disease. Neladenoson, an A_1_R agonist and capadenoson derivative, has demonstrated to be safe but not effective in HF trials (98). The design of new hA_1_AR partial agonists is becoming a relevant research objective. N^6^-cyclopentyladenosine (CPA), a new A_1_R agonist, decreased the H_2_O_2_-induced intracellular and mitochondrial ROS origin and cellular apoptosis (99).

Elamipretide, studied both to maintain cellular biogenetics and prevent ROS-induced cell injury, has shown to increase LVEF and prevent LV remodeling in animal models of HFrEF. In clinical trials, elamipretide was not associated to any serious adverse events (9), with promising improvements in cardiac hemodynamics at highest doses (100).

Between recent discoveries in pathophysiology of HF, mitochondria-induced cell death seems to play a pivotal role. Thus, identification of mitochondria-related genes (Mito-RGs) based on transcriptome sequencing data may be useful as potential new markers and new pharmacological targets for HF (101).

Mitochondrial complexes are known as important mediators for the regulation of cardiomyocyte function. NADH: ubiquinone oxidoreductase core subunit S1 (Ndufs1) expression is reduced in the HF. Cardiac-specific Ndufs1 overexpression improves cardiac function and decreases myocardial fibrosis. Being able to modulate cardiac function, Ndufs1 could be a possible pharmacological target in patients with cardiac dysfunction or HF.

In experimental HF, Empagliflozin stimulate cardiac mitochondrial function and upregulated energy metabolism (102). Empagliflozin improve mitochondrial biogenesis, enhancing mitochondrial oxidative phosphorylation (OXPHOS), reducing ROS production, attenuating apoptosis, and increasing autophagy (103). Empagliflozin is able to modulate the expression of mitochondrial fission-related proteins and mitochondrial fusion-related proteins, in different way, upregulating the expression of the fusion-related proteins and downregulating fission-related proteins (104).

The antioxidant mitoquinone (MitoQ) may affect the production of mitochondrial ROS and hypertrophy in rat cardiomyocytes (105). MitoQ in cardiomyocytes breaks up the metabolism by impaired mitophagy, inducing to accumulation of deficient mitochondria. A Phase II, double blind, randomized trial of MitoQ in patients with DCM, evaluating the effect of reducing mitochondrial oxidative stress on myocardial energetics and function by cardiac magnetic resonance, was planned (13).

Mitochondrial Ca^2+^ handling may be an interesting target in HF patients because its modulation play a key role to the energy supply of the heart contractions as well as to avoiding mitochondrial Ca^2+^ overload and the cell death induction. In models damaged mitochondrial Ca^2+^ transport is related to the initiation/progression of HF syndrome. Mitochondrial Ca^2+^ uptake is regulated by the Ca^2+^ uniporter (mtCU) and the MCU pore is regulated by the Ca^2+^-sensing MICU1 and MICU2 (106). We suggest a possible pharmacological target at the level of the MICU1-dependent regulation of the mtCU. Mitochondria-localized circular RNAs (circRNAs) are able to regulate mitochondria-derived ROS production. Recently, Hao Zheng et al. suggested that circSamd4 (generated by circularization of exon 3 of the Samd4 gene located on chromosome 14 (mm9) is a new pharmacological target in case of HF after MI (107).

The endoplasmic reticulum (ER) and mitochondria are associated by ER-mitochondria contacts (ERMCs). It may contribute to preserve the normal cellular function and may be a new target for potentials drugs against cardiac remodeling in HF (108).

Remote ischemic preconditioning (RIPC) significantly reduces the *rate* of acute kidney injury in non-diabetic *subjects* undergoing PCI. The RIPC stimulus leads release of messengers which act by the bloodstream resulting in *decreased* oxidative stress and preservation of mitochondrial function (109).

RIPC may be considered as drugs using mitochondria as target (110).

## 5. Drugs acting on intracellular calcium dysregulation

Intracellular calcium dysregulation is an important feature of cardiomyocytes in HF (111). In fact, in this condition, the sarcoplasmic reticulum of these cells has a poor calcium content, partly as a result of a reduced SERCA isoform 2a pump activity (112). Istaroxime, a new HF drug, acts through a dual pathway: Na^+^/K^+^ ATPase blockage and potentiation of Ca^2+^ uptake into sarcoplasmic reticulum (SR) mediated by the SERCA2a. This is at the basis of its inotropic and lusitropic effects, due to the ability of dissociating SERCA from its inhibitory protein phospholamban with a lower risk of Ca^2+^ triggered arrhythmias (113).

The phospholamban is an important regulator of cardiac contractility. Interfering with the phospholamban/SR Ca^2+^ ATPase interaction *might* be a promising *pharmacological* approach for HF. Phospholamban-mediated SERCA2a activity *enhancement* may *lead an improvement of* cardiac function. This action may be explained by the activation of the proteinkinase A (PKA). Approaches to selectively inhibit phospholamban remain elusive (114).

The mutations in phospholamban *decrease* its phosphorylation level by *modifying* its conformation and weakening its interactions with PKA (115). *Most of* mutations identified in phospholamban have been *demonstrated to be related* to familial DCM.

In the HORIZON-HF trial 120 patients with acute HF have been treated with intravenous istaroxime or placebo in a 3:1 ratio. All three dosages of istaroxime were associated with a decrease in pulmonary capillary wedge pressure, but cardiac index increased only at the highest dose, that also was linked to a lusitropic effect assessed by increased mitral deceleration time. Moreover, these effects were accompanied by an increase systolic blood pressure but not in heart rate, unlike traditional inotropes (10). In the chronic setting of HF the genetic modulation of SERCA 2a is arousing increasing interest. The CUPID 2 (The Calcium Up regulation by Percutaneous Administration of Gene Therapy in Cardiac Disease) study has examined the efficacy of gene transfer using adenovirus as vectors. Patients receiving the high dose of SERCA2a complementary DNA by intracoronary infusion had satisfactory outcomes as regards symptoms and NT-proBNP values. After 1 year, researchers registered a significant increase in time to clinical events, and faster CV hospitalizations with the high-dose treatment versus placebo and, after 36 months, a reduction in the risk of recurrent CV events by 82%, together with the evidence of long-term transgene presence. CUPID approach should be replicated in broader studies, but SERCA2a gene therapy is definitely appealing in the pharmacological armamentarium against HF (116). The MUSIC-HFrEF1 ongoing trial is evaluating the efficacy and safety of the modulation of SERCA2a by gene therapy in patients with HFrEF and NYHA class III/IV (14).

The ryanodine receptors of the SR cause release of calcium even in the active state and, under resting condition, are maintained closed by a protein said calstabin-2. The continuous adrenergic activation in HF causes a chronic cAMP-dependent hyperphosphorylation of these receptors. Thereby the ryanodine receptor channel opens inappropriately during the diastole, with a consequential calcium leakage from sarcoplasmic reticulum. The loss of calcium from the SR causes weak cardiac contractions. Minimizing the calcium leak by stabilizing the interaction between ryanodine receptor and calstabin could be a viable strategy for improving cardiac contractility (109). JTV519 (K201) is a drug consolidating the closed state of ryanodine receptor by improving its affinity to calstabin, thus preventing spontaneous diastolic calcium leakage (109). In animal myocardial infarction model and pacing-induced HF, K201 has been shown to attenuate significantly the development of cardiac dysfunction and ventricular remodelling (117).

With further studies, probably in the future this drug could be used for some human myocardial diseases.

1. *In vitro* studies have showed the inotropic and lusitropic effects of nitroxyl (HNO) donors that act increasing calcium sensitivity and calcium handling efficiency. HNO-induced increase of myocardial Ca^2+^ cycling and function is due to the post-translational adaptation of thiol residues belonging to proteins like SERCA2a, ryanodine receptors, phospholamban and myofilaments (118).
2. HNO also induces a peripheral vasodilation through the endothelial soluble guanylate cyclase but does not cause tachyphylaxis, unlike the chemically related nitric oxide (119).

The STAND-UP AHF trial evaluated the effects of cimlanod (a HNO donor) in patients hospitalized for AHF. Its intravenous infusion may reduce systemic pressure and intra-cardiac filling pressures, and may increase cardiac index (CI) (118), but these did not last beyond the end of treatment (119). Moreover Lang et al. hypothesized that the CI improvement could come from a vasodilatory effect rather than to an inotropic property of cimlanod. The StandUP-Imaging trial was designed to evaluate the effects of cimlanod infusion on cardiac performance, evaluated through an invasive assessment and echocardiographic parameters, in patients affected by HFrEF and in stable clinical conditions. Nitroglicerin (NTG), a vasodilator without direct inotropic effects, was an active comparator. In this cohort of patients cimlanod did not exert inotropic and lusitropic effects, performing haemodynamic effects similar to NTG. Both improved diastolic function and this could be explained by venodilatation and preload decrease rather than by a lusitropic effect. Ongoing trial will better define its role in the treatment of HF (118).

Another new possible target in HF therapy may be the Na^+^/H^+^ exchanger-1 (NHE-1). Myocytes in HF show an upregulated activity of this exchanger and an increased intracellular concentration of Na^+^, with a consequent increment of intracellular Ca^+^. As it is known an abnormal calcium handling is linked to cardiac hypertrophy and remodelling (120). NHE-1 is also involved in the endoplasmic reticulum stress-induced apoptosis and NHE-inhibition may prevent/slow down cell necrosis, stunning, reperfusion arrhythmias and mortality in different models of ischemia/reperfusion damage (121). Increasing evidence suggests that NHE-1 inhibitors such as cariporide, eniporide and zoniporide may protect against cardiac hypertrophy, ischaemia/reperfusion injury, and myocardial infarction. Cariporide moreover, demonstrated to be able to reduce left ventricular end-systolic volumes and to increase the LVEF, when administered intravenously before a percutaneous coronary intervention in patients affected by acute myocardial infarction. Other strategies to inhibit NHE-1 could include microRNAs and engineered dominant-negative components, small molecules that inhibit signaling kinases downstream to NHE-1 (122). Although a possible target for a pharmacological approach in cardioprotection, NHE-1 is also ubiquitously expressed and play a role in normal cellular pathophysiology and its inhibition may lead in important adverse effects. For example, NHE1 favors platelet aggregation and in the EXPEDITION study the cardioprotective effect of cariporide has been obscured by an increase in ischemic stroke (122).

## 6. Drugs acting on cardiac myosin

Multiple saving-life therapies act only on neurohormonal activation in HFrEF, without addressing the central pathogenic driver: the aforementioned initial reduction in systolic function (11). Myosin modulators are a new class of molecules that directly stimulate contractility and cardiac power output, to relieve HF symptoms without altering calcium signaling and without the side effects of traditional inotropic agents (123).

Omecamtiv Mecarbil (OM) is a selective cardiac myosin activator that increase myocardial contractility by binding myosin when myosin-binding sites on actin filaments are blocked by tropomyosin. Calcium allows tropomyosin to occasionally reveal the “open” state of actin. OM-bound myosin rapidly releases a molecule of Pi and binds the actin filament. This maintains the actin filament in the open phase, allowing non-OM-bound myosin to attach themselves to actin (123). The process results in a higher number of “force generators” (myosin heads) that can bind to actin, developing a higher-force of contraction at a given calcium concentration. By stabilizing the pre-power stroke phase, OM also reduces the ATP turnover and increases the ATP availability for any other energetic scope (11). Studying the OM effects with echo, searchers registered an improvement of systolic ejection time (SET) and stroke volume (SV) with a decrease in LV diameters and volumes (124).

GALACTIC-HF, a multicenter, double-blind, phase 3 trial, compared OM versus placebo among about 8,000 subjects affected by HF (NYHA functional class II-IV) and LVEF ≤35%, who were randomized on top of a standard of HF therapy. Unlike other drugs specific for HFrEF, omecamtiv mecarbil did not affect blood pressure, renal function or potassium levels. Patients classified as having severe HF (NYHA III-IV, LVEF < 30%, last hospitalization within 6 months) experienced a greater benefit from OM than those without severe HF. For the primary end point (time to first HF event or CV death), they showed a 20% risk reduction, whereas patients without severe HF did not show relevant risk reduction. Prolonged (20 weeks) treatment with OM was also associated with serum troponin increase in the phase II study COSMIC-HF (124) while in the GALACTIC-HF patients with severe HF experienced a greater rate of cardiac infarction, expecially if their HF had an ischaemic origin (125). The long-term advantages of this new treatment is currently unknown. To sum up cardiac myosin activators could be an attractive therapeutic option, especially for subjects with lower blood pressure values, reduced kidney function, lower LVEF values and at high risk of recurrent hospitalization or advanced HF (125). These data support the potential role of OM in subjects for whom actual pharmacological possibility are slim or not sufficient (11). However, patients in atrial fibrillation and flutter at baseline were less likely to benefit from OM than subjects without arrhythmias as atrial fibrillation and flutter, although the reduction of the advantages was especially noticed in subjects affected by HF and atrial fibrillation or flutter who were also receiving digoxin (126).

Danicamtiv, another myosin activator, plays similar effects on cardiac myosin. In a phase 2a trial on patients with a stable chronic HF and a LVEF ≤ 35%, already treated according guideline medical therapy, it prolonged the SET and, as a consequence, improved SV and reduced LV dimensions. Other echocardiographic measures were positively affected by Danicamtiv: LV global longitudinal and circumferential strain, left atrial (LA) minimal volume, LA emptying fraction (LAEF) and LA function index (LAFI). The drug seems quite tolerated with small and asymptomatic fluctuations in troponin values demonstrated in the minority of subjects (127). A phase 2a study aiming at establishing safety and preliminary efficacy of danicamtiv in patients with idiopatic DCM due to myosin 7 (MYH7) or titin (TTN) variants was designed (16).

Mavacamten is a new, oral, inhibitor of cardiac myosin ATPase, which *reduces* the formation of actin- myosin cross-bridges, reducing myocardial contractility, and improving myocardial energetics. *It was* approved by the US Food and Drug Administration for the treatment of symptomatic obstructive hypertrophic cardiomyopathy (*NYHA functional* class II to III) in adults to improve functional capacity and symptoms (128).

## 7. Conclusions

New HF drugs might target myocytes and interstitium, cardiac metabolism, mitochondrial function and intracellular calcium dysregulation. New studies are needed to show new potential pharmacological strategies aimed at new pharmacological targets in HF.
